# Chronic exposure to haloperidol and olanzapine leads to common and divergent shape changes in the rat hippocampus in the absence of grey-matter volume loss

**DOI:** 10.1017/S0033291716001768

**Published:** 2016-08-12

**Authors:** W. R. Crum, F. Danckaers, T. Huysmans, M.-C. Cotel, S. Natesan, M. M. Modo, J. Sijbers, S. C. R. Williams, S. Kapur, A. C. Vernon

**Affiliations:** 1 Department of Neuroimaging, King's College London, Institute of Psychiatry, Psychology and Neuroscience, Centre for Neuroimaging Sciences, De Crespigny Park, London, UK; 2 Department of Physics, iMinds-Vision Laboratory, University of Antwerp, Antwerp, Belgium; 3 Department of Psychosis Studies, King's College London, Institute of Psychiatry, Psychology and Neuroscience, De Crespigny Park, London, UK; 4 Department of Basic and Clinical Neuroscience, King's College London, Institute of Psychiatry, Psychology and Neuroscience, Maurice Wohl Institute for Clinical Neuroscience, London, UK

**Keywords:** antipsychotic, hippocampus, magnetic resonance imaging, schizophrenia, shape, volume

## Abstract

**Background:**

One of the most consistently reported brain abnormalities in schizophrenia (SCZ) is decreased volume and shape deformation of the hippocampus. However, the potential contribution of chronic antipsychotic medication exposure to these phenomena remains unclear.

**Method:**

We examined the effect of chronic exposure (8 weeks) to clinically relevant doses of either haloperidol (HAL) or olanzapine (OLZ) on adult rat hippocampal volume and shape using *ex vivo* structural MRI with the brain retained inside the cranium to prevent distortions due to dissection, followed by tensor-based morphometry (TBM) and elastic surface-based shape deformation analysis. The volume of the hippocampus was also measured post-mortem from brain tissue sections in each group.

**Results:**

Chronic exposure to either HAL or OLZ had no effect on the volume of the hippocampus, even at exploratory thresholds, which was confirmed post-mortem. In contrast, shape deformation analysis revealed that chronic HAL and OLZ exposure lead to both common and divergent shape deformations (*q* = 0.05, FDR-corrected) in the rat hippocampus. In particular, in the dorsal hippocampus, HAL exposure led to inward shape deformation, whereas OLZ exposure led to outward shape deformation. Interestingly, outward shape deformations that were common to both drugs occurred in the ventral hippocampus. These effects remained significant after controlling for hippocampal volume suggesting true shape changes.

**Conclusions:**

Chronic exposure to either HAL or OLZ leads to both common and divergent effects on rat hippocampal shape in the absence of volume change. The implications of these findings for the clinic are discussed.

## Introduction

Large-scale magnetic resonance imaging (MRI) studies of patients with schizophrenia (SCZ) commonly report decreases in hippocampal volume with robust effect sizes (van Erp *et al.* 2015). Advances in computational neuroanatomical techniques have refined this to include descriptions of progressive shape deformations that also occur in the hippocampus of SCZ  patients (Csernansky *et al.* 1998, 2002; Wang *et al.* 2001; Shenton *et al.* 2002; Zierhut *et al.* 2013; Mamah *et al.* 2016). However, brain differences detected by MRI are influenced by multiple factors, some with potentially opposing effects, including the duration of exposure to antipsychotic medication (Lieberman *et al.* 2005; Ho *et al.* 2011). Dissecting the effects of disease and other moderating factors, such as medication exposure in clinical MRI studies is therefore extremely difficult.

Data from the ENIGMA Schizophrenia Working Group (*n* = 2028 SCZ patients and *n* = 2540 controls) suggests that decreases in hippocampal volume are positively associated with the proportion of unmedicated SCZ patients (van Erp *et al.* 2015). This suggests that structural abnormalities of the hippocampus may be more severe in untreated patients and potentially, those volume deficits may be ameliorated, at least partially, by treatment with antipsychotic drugs (APD) (Szeszko *et al.* 2003; Narr *et al.* 2004; van Erp *et al.* 2015). In the recent ENIGMA dataset, 11 of the study sample sites included patients predominantly treated with second-generation (atypical) antipsychotics (SGA) (van Erp *et al.* 2015). Importantly, longitudinal MRI studies, which have the advantage of controlling for baseline changes prior to medication exposure, report significant decreases from baseline in either the grey-matter volume, or the shape of the temporal lobe and the hippocampus in SCZ patients treated with first-generation antipsychotics (FGA), but not SGA (Lieberman *et al.* 2005; McClure *et al.* 2006, 2008, 2013; Koolschijn *et al.* 2010). A more recent study replicated the finding of reduced temporal lobe grey-matter volume, but this effect was independent of APD treatment (Ho *et al.* 2011). In addition, higher doses of quetiapine are associated with decreased hippocampal grey-matter volume (Ebdrup *et al.* 2011). In parallel to volume studies, longitudinal analysis of shape deformations in the hippocampus reveals that these are present in ultra-high-risk individuals and persist following transition to psychosis (Dean *et al.* 2016). Furthermore, inward shape deformation of the posterior hippocampus appears to predict the degree of positive symptoms in these patients (Dean *et al.* 2016). Longitudinal studies have also attempted to examine the potential influence of antipsychotics on hippocampal shape. Mamah *et al*. (2012) report progressive hippocampal shape abnormalities between SCZ patients treated with haloperidol (HAL), compared to olanzapine (OLZ) (Mamah *et al.* 2012). Shape deformation of the CA1 region in SCZ patients is also reported to predict the dose of APD required to treat positive symptoms (Zierhut *et al.* 2013). Conversely, at least four longitudinal studies report no significant relationships between antipsychotic use and hippocampal volume or shape (Arango *et al.* 2003; Velakoulis *et al.* 2006; Panenka *et al.* 2007; McClure *et al.* 2013). However, the lack of longitudinally followed untreated patients as a control means it remains unclear whether these outcomes are the effect of illness progression, antipsychotic treatment or an interaction between the two. Further, none of the human studies have linked the imaging changes to post-mortem findings and therefore the relationship between imaging-related structural changes and post-mortem findings remains unclear (Vernon *et al.* 2011). Thus, whilst exposure to APD have been linked to both hippocampal volume and shape changes in SCZ patients, the findings are clearly equivocal.

Pre-clinical studies incorporating rodent MRI have previously been useful to address this issue, providing evidence that chronic exposure to either HAL or OLZ leads to alterations in brain volume (Vernon *et al.* 2011, 2012, 2014). To date, however, there has been no investigation of the effect of chronic APD exposure on shape metrics, although this is possible using rodent MR images (Delgado y Palacios *et al.* 2011; Wheeler *et al.* 2013). In the current study, we set out to address this question by utilizing *ex vivo* MRI data available from our prior study of the effects of chronic exposure to APD on rat brain volume (Vernon *et al.* 2011). Specifically, we tested for the presence of hippocampal volume differences per voxel, using tensor-based morphometry (TBM) in male rats exposed for 8 weeks to either HAL (2 mg/kg per day) or OLZ (10 mg/kg per day), compared to vehicle (VEH)-treated controls. TBM is a sensitive and observer-independent measure of brain atrophy, which is well suited to rodent MRI studies where the grey- to white- matter ratio is low (Lau *et al.* 2008; Lerch *et al.* 2008; Vernon *et al.* 2014; Harrison *et al.* 2015). In parallel, we applied a novel elastic surface-based shape analysis method to search for deformations in hippocampal shape as a function of chronic APD exposure. Based on our prior observations that chronic exposure to either HAL or OLZ did not lead to a decrease in hippocampal volume, using manual segmentation analysis, we hypothesized that the more sensitive TBM analysis may potentially disprove this finding. In terms of shape metrics, since the available clinical data are equivocal, we made no specific *a priori* hypothesis.

## Materials and method

### Animals

This study used *ex vivo* MR images and brain tissue sections collected from VEH- and APD-exposed animals, as previously reported (Vernon *et al.* 2011) (see also Supplementary materials and methods). No new animals were generated for this study. Briefly, a common VEH (*β*-hydroxypropylcyclodextrin, 20% w/v, acidified by ascorbic acid to pH 6; *n* = 8), HAL (2 mg/kg per day; *n* = 8; Sigma-Aldrich, UK), or OLZ (10 mg/kg per day; *n* = 8; Biophore Pharmaceuticals Ltd, India) were administered to experimentally naive, 10-week-old male Sprague–Dawley rats (Charles River, UK), using subcutaneously implanted osmotic minipumps for a total of 8 weeks (Vernon *et al.* 2011). Osmotic minipumps (Alzet Model 2ML4, 28 days; Alzet, USA) filled with drug or VEH solutions were inserted subcutaneously on the back flank under isoflurane anaesthesia (5% induction, 1.5% maintenance) and replaced once after 28 days. Animals were habituated for 7 days before experimental procedures, which were carried out in accordance with the Home Office Animals (Scientific procedures) Act, United Kingdom, 1986 and European Union Directive 2010/63/EU.

### MRI acquisition

Brains were prepared for *ex vivo* MR imaging as previously described (Vernon *et al.* 2011, 2014) (see also Supplementary materials and methods). Briefly, MR image acquisition was performed using a 7 T horizontal small bore magnet (Varian, USA) with custom-made quadrature volume radiofrequency (RF) coil (43 mm inner diameter, Rapid Biomedical GmbH, Germany) connected to a console running VnmrJ acquisition software (v. 2.3; Varian). A modified multiecho, multislice spin-echo pulse sequence was used for image acquisition, with the following parameters: field of view = 35 × 35 mm^2^; matrix = 192 × 192; repetition time = 4200 ms; echo time = 10, 20, 30, 40, 50, 60, 70, 80 ms; 8 averages, 50 slices, 0.5 mm thick, with a total duration of 2 h 30 min and an in plane resolution of 187 μm.

### Assessment of hippocampal volume using TBM

**Fig. 1.**
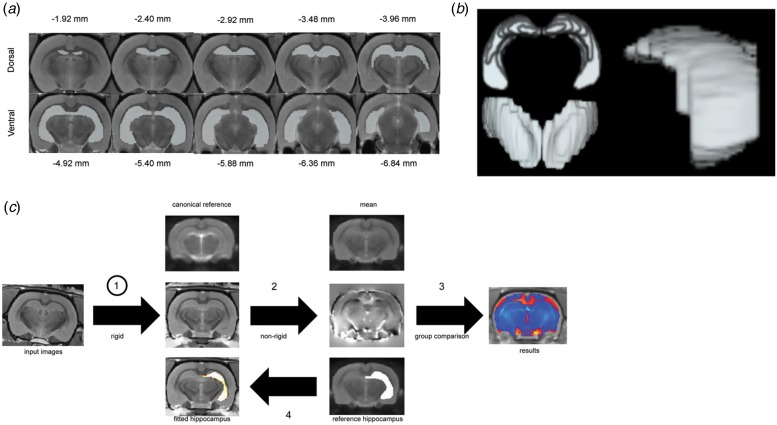
(*a*) Anatomical criteria for creation of the binary mask for tensor-based morphometry (TBM) and shape analysis of the rat hippocampus using a mean MR image of the entire dataset (*n* = 24 scans). The orientation of the displayed scans is in the coronal plane. (*b*) Three-dimensional rendering of the binary mask of the rat hippocampus. (*c*) Processing pipeline for automated TBM analysis.

A mean image of the entire dataset (*n* = 24 scans) was generated using rigid-body registration (6 d.f.) using a population-based registration method based on FSL-FLIRT (Jenkinson & Smith, 2001; Jenkinson *et al.* 2002; Crum *et al.* 2013). Using this mean image, the external and internal borders of the left and right hippocampus (dorsal and ventral regions, approximately to −1.92 to −6.84 mm from bregma based on the rat stereotaxic atlas (Watson & Paxinos, 2007) were manually defined using the polygon tool in ITK-SNAP (http://www.itksnap.org) (Yushkevich *et al.* 2006) by an expert rater (A.C.V.) using previously published criteria (Wolf *et al.* 2002; Vernon *et al.* 2011) (Fig.1*a, b*). Segmentation performance was assessed using intra-class correlation coefficient with values <0.95 rejected. This segmentation was used to create a binary mask for implementation in the TBM pipeline (Fig. 1*c*). Tensor-based morphometry was used to assess anatomical differences related to APD treatment in the hippocampus as previously described (Vernon *et al.* 2014) (see also Supplementary materials and methods). Using high-dimensional non-rigid fluid registration, the binary hippocampus mask defined in the template space was warped back onto individual MR images using the inverse of the transformation and used for subsequent shape analysis.

### Assessment of hippocampal shape using elastic surface-based shape deformation analysis

**Fig. 2.**
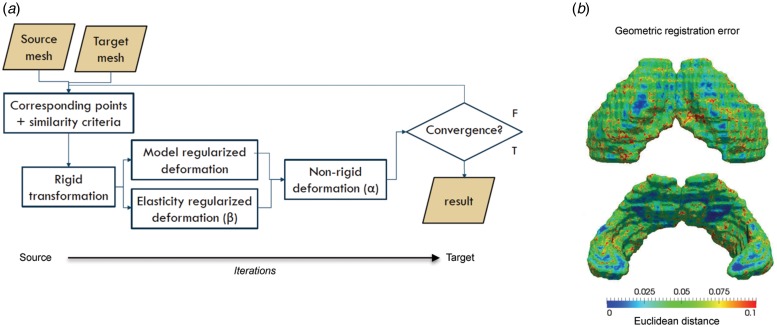
(*a*). Image processing pipeline for elastic surface-based shape deformation analysis. (*b*) The geometric error (Euclidean distance) between the original surface and the registered surface. This error is calculated for every surface and the average error is projected on the average surface and displayed with a colour map.

The shape analysis pipeline has been reported previously (Danckaers *et al.* 2014) and is depicted in Fig. 2*a*. Changes in the shape and size of the hippocampus may be described by computing the inward and outward displacement vectors from the surface of the structure (Delgado y Palacios *et al.* 2011; Wheeler *et al.* 2013). First, a reference hippocampal surface is registered to a target hippocampal surface, by minimizing the geometric distance between those surfaces while maintaining correspondences. Here, the binary hippocampus mask (reference) is warped to match the population average image of all rats (target). Fig. 2*b* shows the geometric error (Euclidean distance) between the original surface and the registered surface, calculated for every surface and the average error projected on to the average surface. From this final nonlinear atlas, a surface representation of the hippocampus is generated. Due to the anisotropy resulting from the 2D acquisition (in-slice *v*. axial resolution), the resulting 3D hippocampal surfaces exhibit a stair-like structure. Therefore, in order to obtain a more accurate 3D surface while still interpolating the actual slices, the surfaces were prepared by interpolating the distance transform of adjacent slices over a regular grid. The global rigid registration and an elasticity-modulated registration are then iteratively repeated (*n* = 60 iterations). During the iterations, the stiffness gradually decreases, such that the surface will become more elastic through the iterations. The first step of surface registration is the application of a rigid alignment. To that end, in both hippocampal surfaces corresponding points are identified, by casting a normal ray from each vertex of the reference surface to the target surface. When the normal of an intersection point is in the same direction, within a tolerance of 60°, as the normal of the point on the reference surface, that point can be considered corresponding. This tolerance is based on the dot product between the normal of the source vertex and the intersection of this normal with the target surface. If this dot product >0.5, those points are considered as corresponding points. Another restriction for corresponding points is that the normal may not intersect the surface multiple times before reaching the corresponding point. Once corresponding point sets are obtained, they are used to rigidly align the surfaces in a least squares sense using singular value decomposition.

In the elastic part of the registration, the vertices are allowed to translate separately, while motion is restricted by a stiffness parameter, which regulates the strength of the connection with the neighbouring vertices and which decreases during the iterations. The stiffness parameter is *β* in the pipeline (Fig. 2*a*) and regulates how stiff or elastic the surface can behave. When this parameter is high, the neighbouring vertices of a translated vertex are forced to move (partially) along. In contrast, when this parameter is low, the surface is more elastic and the connection between neighbouring vertices is lower, such that these may move more freely. By applying weights to each vertex, the importance of this vertex can be set. If no corresponding point for a vertex of the source mesh can be found, its weight is set to zero. In that case, this vertex simply translates along with its neighbouring vertices. When all surfaces are registered by the same reference surface, each vertex has the same anatomical location on each hippocampal surface.

### Post-mortem tissue handling and Cavalieri probe analysis of hippocampal volume

After MRI acquisition, brains were removed from the skull and processed for Nissl staining as previously described (Vernon *et al.* 2011) (see also Supplementary materials and methods). A single observer (M.C.C.) blinded to experimental group by coding measured the volume of the hippocampus using the Cavalieri estimator (Gundersen & Jensen, 1987) as described elsewhere (Vernon *et al.* 2011) (see also Supplementary materials and methods).

### Statistical analysis

Voxel-wise analyses of local volume changes for group differences were computed using a one-way ANOVA followed by voxel-wise *t* tests to compare VEH to each antipsychotic drug (VEH *v*. HAL; VEH *v*. OLZ), as well as each antipsychotic to the other (HAL *v*. OLZ) (Vernon *et al.* 2014). Shape changes were analysed per vertex by comparing the vector lengths and direction, performing a covariance calculation using non-parametric permutation tests based on Hoteling's T2 statistics. For both volume and shape analysis, multiple comparisons were controlled for using the FDR at *q* = 0.05 (Genovese *et al.* 2002). Due to the small sample size in the current study, the data were also analysed at an exploratory threshold of *p* < 0.05 uncorrected for multiple comparisons. Post-mortem volume data were analysed using one-way ANOVA with Bonferroni *post-hoc* test using SPSS v. 22.0 software (IBM Corp., USA).

## Ethical standards

The authors assert that all procedures contributing to this work comply with the ethical standards of the relevant national and institutional guides on the care and use of laboratory animals.

## Results

### Chronic APD exposure has no significant effect on hippocampal volume in naive rats

The TBM analysis revealed no significant effects (FDR, *q* = 0.05) of chronic exposure (8 weeks) to either HAL or OLZ on hippocampal volume, in either the left or right brain hemisphere (Supplementary Fig. S1A, B). This replicates our previous null findings using manual segmentation (Vernon *et al.* 2011). As the sample size was small, which may compromise our ability to detect subtle changes, we carried out an additional exploratory analysis (*p* < 0.05 uncorrected; (Vernon *et al.* 2014). However, even at this exploratory threshold we could not detect any clusters of contracted or expanded voxels in our hippocampal region of interest (data not shown).

### Impact of chronic APD exposure on hippocampal shape deformation

**Fig. 3.**
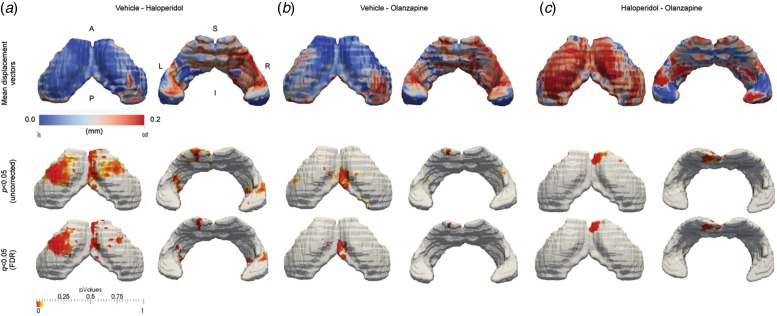
Elastic shape deformation analysis reveals common and divergent effects of chronic haloperidol and olanzapine exposure on hippocampal shape metrics. Data shown are the mean inward and outward displacement vectors (mm), shown both at an exploratory threshold of *p* < 0.05 uncorrected for multiple comparisons and *q* = 0.05 FDR-corrected, to illustrate significant differences in hippocampal shape metrics between (*a*) vehicle and haloperidol; (*b*) vehicle and olanzapine and (*c*) haloperidol and olanzapine exposed rats. Directionality of statistically significant hippocampal shape deformations, either inward (blue) or outward (red), may be found by comparing the *p* value maps with the mean displacement vector maps in each treatment group comparison. A, Anterior; P, posterior; S, superior; I, inferior; L, left; R, right.

The results of the shape analysis are shown in Fig. 3*a*–*c*. It is clear from the data that there are both net outward and inward vertex displacements in hippocampal shape following chronic exposure to either antipsychotic compared to VEH-treated animals. Chronic HAL exposure resulted in significant (*q* < 0.05, FDR-corrected; Fig. 3*a*) inward displacement in the dorsal hippocampus compared to VEH-treated rats. These displacements are bilateral, but appear more pronounced in the left hemisphere of the brain. These inward displacements correspond approximately to the cornu ammonis 1 (CA) and sub-field, extending through the oriens, pyramidal and radiatum layers to touch the CA3 sub-field. Additional significant, bilateral inward deformations were also found in the CA3 sub-field in the ventral hippocampus. In contrast, significant outward deformations were observed in both hemispheres of the ventral hippocampus. These are again bilateral, but predominate in the right hemisphere and correspond approximately to the CA1 and CA2 sub-fields.

Chronic OLZ exposure also led to significant (*q* < 0.05; FDR-corrected; Fig. 3*b*) deformations in the dorsal and ventral hippocampus compared to VEH-exposed rats. Although these deformations overlap topographically with those observed in HAL-exposed animals, specifically to the left dorsal CA1 sub-field and right ventral CA1 and CA2 sub-fields, they are less widespread (Fig. 3*a, b*). Furthermore, the dorsal CA1 deformations in OLZ-exposed animals are in the opposite direction to those in HAL-exposed animals, moving outward, rather than inward, relative to VEH controls (Fig. 3*a, b*). When directly comparing shape differences between HAL- and OLZ-exposed animals, this effect shows up as a subtle, but significant difference between the two APD (Fig. 3*c*). Interestingly however, in the ventral aspect of the hippocampus in OLZ-exposed rats, there is vertex displacement that overlaps both in terms of topography and direction of change with those observed in HAL-exposed animals, although these did not survive multiple comparisons correction (Fig. 3*b*).

**Fig. 4.**
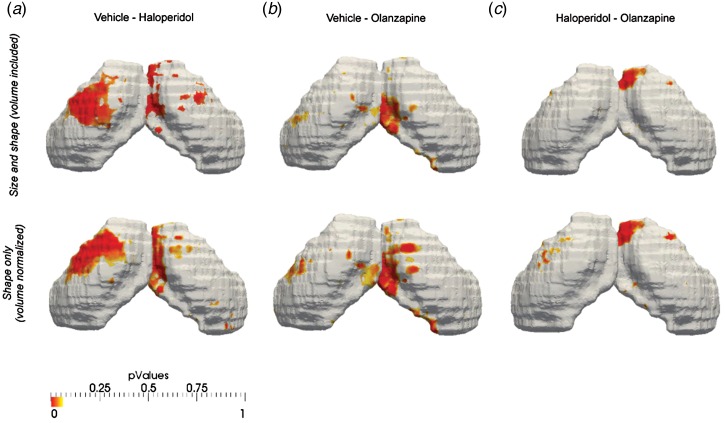
Normalization of hippocampal volume does not alter the pattern of significant voxels identified by elastic shape deformation analysis, suggesting true shape changes. Data shown are size and shape and shape only (volume normalized to 1) for (*a*) vehicle *v*. haloperidol; (*b*) vehicle *v*. olanzapine and (*c*) haloperidol *v*. olanzapine.

The lack of TBM changes strongly suggests the volume of the hippocampus is not altered across treatment groups and that these deformations represent true shape changes. However, this can also be tested directly using volume normalization in order to visualize the pure shape differences. In Fig. 4, the volume of the hippocampal surfaces is normalized such that each hippocampus has a size of 1. When comparing the data before and after volume normalization, the results are identical, consistent with the lack of volume change detected by TBM or manual segmentation. These data strongly suggest that we are detecting true shape changes in the hippocampus following chronic exposure to APD. The presence of both inward and outward shape deformations is therefore also consistent with the failure to find significant differences in volume, either by TBM or manual segmentation, since the net hippocampal volume change may be very close to zero.

### Confirmation of the absence of hippocampal volume changes post-mortem

**Fig. 5.**
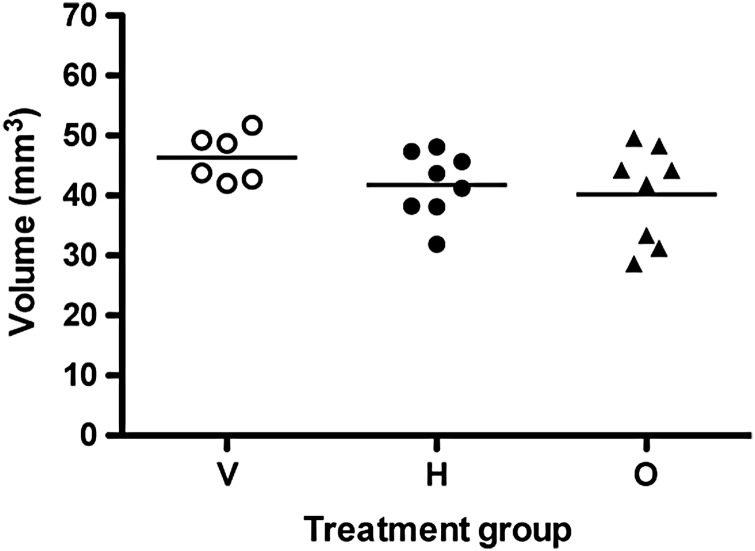
Post-mortem confirmation of the lack of hippocampal volume changes in rats chronically exposed to either haloperidol or olanzapine using the Cavalieri probe. Data shown are individual hippocampal volume (scatter plot with mean) in vehicle (V), haloperidol (H) and olanzapine (O) treated rats.

Post-mortem volume analysis using the Cavalieri probe revealed no significant overall differences in the total volume (dorsal + ventral) of the left hippocampus between treatment groups (*F*_2,21_ = 0.1.75, *p* > 0.05; Fig. 5 and Supplementary Table S1).

## Discussion

Contrary to our hypothesis, TBM analysis confirmed that chronic exposure (8 weeks) to either HAL or OLZ, at clinically relevant doses, does not lead to changes in the volume of the rat hippocampus. In contrast, the results of the shape analysis reveal that chronic exposure to either HAL or OLZ results in common shape changes both in terms of their topography and direction, particularly in the ventral hippocampus. However, the data also provide preliminary evidence for minor divergent shape changes following exposure to these two different antipsychotics, particularly in the CA1 sub-field of the rat dorsal hippocampus. In this region, while the drug-induced shape changes overlap topographically, HAL exposure leads to inward deformations, whilst OLZ leads to outward deformations. Furthermore, hippocampal shape changes appear more widespread following HAL treatment, particularly again in the dorsal hippocampus. These data raise new questions about our interpretation of clinical MRI data and how antipsychotics might impact hippocampal structure in SCZ patients.

In the largest cross-sectional MR imaging dataset available comparing healthy controls and SCZ patients, including both medicated and un-medicated individuals (ENIGMA; van Erp *et al.* 2015), the volume of the hippocampus in SCZ patients was significantly reduced (Cohen's *d* = −0.46). This reduction was more pronounced in patients who were not medicated (van Erp *et al.* 2015). Interestingly, the majority of medicated patients were treated with SGA. Although the FGA/SGA nomenclature is a poorly defined and heterogeneous construct (Leucht *et al.* 2009), prior studies suggest there may be distinct effects of different APD on brain volume. For example cortical volume (Lieberman *et al.* 2005; Ansell *et al.* 2015) and cortical thickness (van Haren *et al.* 2011) are reported to be less apparent in SCZ patients treated with SGA, including OLZ. A recent meta-analysis of longitudinal structural MRI studies in medicated SCZ patients also suggests SGA have less impact on brain volume, compared to FGA, including HAL (Vita *et al.* 2015). In contrast, primate (Dorph-Petersen *et al.* 2005) and rodent (Vernon *et al.* 2011) pre-clinical studies have failed to find significant differences between the effects of HAL and OLZ on brain volume following chronic exposure, including no effect on hippocampal volume, using manual segmentation (Vernon *et al.* 2011). The results of the current study using operator-independent voxel-wise TBM analysis, which has greater sensitivity for subtle anatomical changes confirm our prior *in vivo* data. Furthermore, we corroborate the TBM analysis post-mortem using unbiased stereology analysis of hippocampal volume from Nissl stained tissue sections. The lack of hippocampal volume change following APD exposure may reflect the fact that these are naive animals, which do not replicate pathological conditions relevant to SCZ. Alternatively, since the hippocampal shape deformations are both inward and outward, the net volume change may be close to zero. These data are, however, consistent with the majority of hippocampal volume MRI studies in patients with SCZ which find no relationship between antipsychotic dose and hippocampal volume (Marsh *et al.* 1994; Whitworth *et al.* 1998; Stefanis *et al.* 1999; Altshuler *et al.* 2000; Gur *et al.* 2000; Arango *et al.* 2003; Szeszko *et al.* 2003; Narr *et al.* 2004; Lieberman *et al.* 2005; Velakoulis *et al.* 2006; Panenka *et al.* 2007; Koolschijn *et al.* 2010; Ho *et al.* 2011).

Importantly, shape analysis complements volumetric analysis and can identify subtle regional abnormalities in brain structures in the absence of overall volumetric changes (Csernansky *et al.* 1998; Csernansky *et al.* 2002). The current study therefore provides the first preclinical evidence that chronic exposure to HAL or OLZ leads to common, but also distinct effects on hippocampal shape. Specifically, chronic treatment with both drugs led to topographically overlapping shape deformations, which were clearly more extensive in HAL than OLZ treated animals. Interestingly, in the dorsal CA1 sub-field, HAL exposure led to *inward* deformation, while OLZ promoted *outward* deformation. In contrast, both HAL and OLZ had similar effects in the ventral hippocampus, promoting predominantly outward deformations, although it is notable these did not survive FDR correction in OLZ-exposed rats. These data are partly consistent with findings from a longitudinal MRI study of first-episode SCZ patients randomized to either HAL or OLZ, which suggested that OLZ treatment was associated with a significantly lower percentage of ‘large magnitude negative surface vertex slopes’ as compared to HAL treatment, when patients were followed up to 104 weeks (Mamah *et al.* 2012). Notably however, other studies on the same dataset found no effect of antipsychotic exposure at any follow-up time point (McClure *et al.* 2006, 2008,  2013). This may be explained by the differential shape analysis methods employed in these studies, which could influence the results (Mamah *et al.* 2016). Our preclinical data suggest the dorsal hippocampus is a potential locus of differential shape changes, whilst the ventral hippocampus shows common shape changes following chronic treatment with either HAL or OLZ in the naive rat brain. Although preliminary, these data suggest a testable hypothesis for future studies with a larger sample size to confirm these data. This could also provide a dataset for the controlled testing of the effects of different shape analysis methodology, which may also be beneficial to the field.

It is conceivable however, that the observed hippocampal shape changes may reflect volume changes in adjacent brain regions, which cause an alteration in hippocampal shape rather than volume. In our prior work, we have shown that chronic antipsychotic exposure decreases total cortical volume, but primarily in the cingulate and somatosensory cortex (Vernon *et al.* 2011, 2014). Chronic HAL exposure also leads to striatal enlargement (Vernon *et al.* 2012). However, as yet we do not have information regarding the effects of chronic antipsychotic exposure on the shape or volume of other sub-cortical nuclei in close proximity to the hippocampus, in particular, the thalamus. Further studies using both TBM and shape analysis are required to assess the potential influence of alterations in volume or shape metrics in these structures on the present findings in the hippocampus.

What might be the cellular origin of these APD-induced hippocampal shape changes? Rigorous post-mortem studies in macaque monkeys using clinically comparable plasma levels, chronic antipsychotic treatment, and unbiased stereology reported a significant 20% decrease in the total number of S100*β* + astrocytes albeit in the grey matter of the left parietal lobe, but no change in neuronal number (Konopaske *et al.* 2008). These provide immediately testable hypothesis for future studies in the rat brain. Using post-mortem brain tissue from the same animals from which the current imaging data were generated, we have recently reported that chronic HAL and OLZ exposure resulted in highly significant increases in the density of amoeboid Iba1+ microglia in both the dorsal and ventral rat hippocampus (Cotel *et al.* 2015). These effects on microglia thus overlap topographically with the areas of shape change in the rat hippocampus after chronic HAL or OLZ exposure. Furthermore, both HAL and OLZ have been previously reported to impact on growth factors, particularly nerve growth factor (NGF), brain-derived neurotrophic factor (BDNF) and fibroblast growth factor (FGF) (Pillai & Mahadik, 2006; Pillai *et al.* 2006). Moreover, oxidative stress in the hippocampus has been reported following HAL, but not OLZ treatment (Reinke *et al.* 2004). Altogether, changes in these parameters could lead to alterations in axon sprouting, fibre reorganization, myelin formation; neurogenesis, dendritic spine morphology (Uranova *et al.* 1991) and angiogenesis, all of which reflect potential contributors to the observed hippocampal shape changes.

The clinical impact of these APD-induced shape changes also remains unclear. Indeed, it is challenging to label the directionality of our findings as ‘beneficial’ or ‘harmful’ (Lewis, 2011). The human (or primate) hippocampus may be functionally divided along its anterioposterior axis into an anterior part, which is involved in emotional processing and a posterior part, which is predominantly involved in memory function. In contrast, the rat hippocampus is orientated along a dorsal-ventral axis, divided into the dorsal and ventral sections, which are also functionally dissociated (Moser & Moser, 1998; Fanselow & Dong, 2010). Specifically, the dorsal part of the rat hippocampus contributes to cognitive functions, including spatial learning and memory and is therefore analogous to the human (or primate) posterior hippocampus (Bannerman *et al.* 2004; McHugh *et al.* 2004). In contrast, the rat ventral hippocampus is involved in the regulation of stress, anxiety and emotional memory and is therefore analogous to the human (or primate) anterior hippocampus (Barkus *et al.* 2010). As such, both divisions of the hippocampus show differential connectivity patterns. For example, memory function is critically dependent upon connections between the dorsal hippocampus and retrosplenial granular and anterior cingulate cortices (ACC) (Fanselow & Dong, 2010). Patients with SCZ exhibit non-specific hyperactivity of the left anterior hippocampus during a long-term memory task, which is related to positive symptom severity (Zierhut *et al.* 2010). Furthermore, ultra-high risk and first-episode psychosis individuals demonstrate increased cerebral blood flow specifically in the anterior CA1 sub-field of the hippocampus, which correlates with atrophy of this region and predicts transition to psychosis (Schobel *et al.* 2009; Schobel *et al.* 2013). Several studies have also identified inward shape deformations in the anterior and posterior aspects of the human hippocampus, which are correlated with the extent of positive symptoms in SCZ patients (Csernansky *et al.* 2002; Small *et al.* 2011; Zierhut *et al.* 2013; Dean *et al.* 2016; Mamah *et al.* 2016). Inward shape deformations of the anterior and posterior CA1 sub-field also predicts daily antipsychotic drug dosage in SCZ patients (Zierhut *et al.* 2013; Dean *et al.* 2016). Shape deformations of the CA1 sub-field, may therefore be the consequence of a disease-specific hyperactive hippocampal state, leading to elevated glutamate levels, downstream atrophy and disruption of CA1 functional connectivity, resulting in the symptomatology of SCZ (Schobel *et al.* 2009; Schobel *et al.* 2013; Zierhut *et al.* 2013). Speculatively, the common outward shape deformations we observe in the ventral hippocampus, the rat homologue of the human anterior hippocampus following chronic exposure to either HAL or OLZ, may reflect a structural consequence of alterations in hippocampus/CA1 activity or functional connectivity, which may be related to the therapeutic effects of these medications on positive symptoms, through correction of aberrant brain activity patterns. In other words, a beneficial effect related to the therapeutic action of the drug.

In contrast, these APD-induced hippocampal shape deformations may be related to adverse motor and cognitive side-effects of APD. Notably, we previously observed common effects of both HAL and OLZ to decrease ACC volume, which were more apparent in HAL-treated rats (Vernon *et al.* 2014). These data may suggest a link between hippocampal shape deformations, ACC volume and functional connectivity of these structures following chronic antipsychotic drug treatment, which requires exploration. Although recent studies have begun to examine the complex network effects of antipsychotics on brain functional connectivity (Gass *et al.* 2013; Wheeler *et al.* 2014), no study has to date linked these to morphometric changes or behavioural outcomes following chronic dosing. Of possible relevance to this study however, Gass *et al*. (2013) reported decreased functional connectivity between the substantia nigra and the CA2 region of the dorsal hippocampus in rats following *acute* HAL challenge (Gass *et al.* 2013). Further studies are required combining functional and structural MR imaging to address whether alterations in brain activity are related to structural remodelling following chronic APD exposure. Importantly, there is a paucity of preclinical studies examining the relationship between chronic APD exposure using clinically relevant dosing and the cognitive or emotional functions ascribed to the dorsal and ventral aspects of the rodent hippocampus. Although prior studies have not typically used mini-pump delivery methods, acute and chronic treatment with various APD leads to impairment in Morris water-maze tasks of spatial learning but not other maze tasks, such as the elevated plus maze or radial arm maze (Skarsfeldt, 1996; Didriksen *et al.* 2006; Terry & Mahadik, 2007; Hutchings *et al.* 2013). Further studies are therefore required to understand the links between dorsal and ventral hippocampus shape deformations, cognitive and emotional behaviour, particularly any differences, or indeed, similarities, following exposure to HAL or OLZ.

It should be noted, however, that we examined male rats only. This is relevant, since a prior study reported a significant decrease in hippocampal volume, following chronic OLZ exposure in female SD rats (Barr *et al.* 2013). Interestingly, this decrease was related to metabolic side effects of chronic OLZ exposure, in this case, impaired glucose tolerance and increased peripheral adiposity (Barr *et al.* 2013). Notably, we have previously reported that the OLZ-exposed animals used in this study have lower hepatic levels of Insulin receptor substrate 2 (IRS2), indicative of impaired insulin signalling and gluco-metabolic abnormalities (Mondelli *et al.* 2013). However, these animals did not show weight gain or increased adiposity as compared to VEH controls (Mondelli *et al.* 2013). In contrast, the HAL-exposed rats showed increase adiposity, in the absence of weight gain and no change in hepatic IRS2 protein levels (Mondelli *et al.* 2013). These data suggest that different molecular pathways mediate the disturbances of glucose homeostasis induced by HAL and OLZ, at least in male rats; nonetheless, metabolic abnormalities were clearly present in both antipsychotic exposed groups. However, female rats produce more consistent and perhaps larger, metabolic changes than males in response to antipsychotic exposure (Boyda *et al.* 2010) which may contribute to reduced hippocampal volume (Barr *et al.* 2013). However, the study by Barr and colleagues also used different dosing methods (daily injection *v*. osmotic minipumps in the current study), which may affect the data by virtue of differential pharmacokinetics (Kapur *et al.* 2003). Nevertheless, investigations into potential sex differences in the effects of APD on brain structure and function, particularly in relation to peripheral metabolic side effects of these drugs are clearly required.

Importantly, the current experiments were conducted in healthy rats, free from pathology, in order to define the effects of chronic antipsychotic exposure on shape and volume metrics in a controlled fashion. Ultimately, however, to begin to establish whether these drug-induced hippocampal shape changes are either beneficial or harmful, will require repetition of these experiments in rat models that model relevant aspects of SCZ pathology. Specifically, these could include MR-detectable increases in hippocampal activity and associated decreases in the volume of the hippocampus such as those observed following either prenatal exposure to maternal infection (Piontkewitz *et al.* 2011) or chronic *N*-methyl-d-aspartate receptor antagonism (Schobel *et al.* 2013; Barnes *et al.* 2015). This may well reveal important differences, including any drug x disease interactions and bring us closer to a clearer vision of the potential clinical implications of these findings. Further caveats regarding the interpretation of our findings should be also be acknowledged. The use of hippocampal surface models relies on manual tracing, and systematic bias in delineating this region might adversely affect its reliability. However, the reliability of the segmentation method has been shown to be high (Wolf *et al.* 2002). Although the tracings were performed blinded to treatment group, any differences in image contrast between VEH and antipsychotic-treated rats could also potentially affect the results. Nevertheless, we have not detected significant changes in T2 signal intensity in other brain regions that displayed volume changes after chronic APD treatment (Vernon *et al.* 2011). The resolution of our MR images is relatively low and the original images were not isotropic. It is therefore possible that partial volume effects or imperfections in image registration may be driving the observed effects of HAL and OLZ on hippocampal shape. Although the registration error between the source and target images was low (see Fig. 2*b*), it will be important to confirm our observations by collecting higher resolution *ex vivo* MR images with isotropic voxels from VEH and antipsychotic-treated rats. The sample size is small (*n* = 8 per group) and our findings thus require confirmation in a larger sample size. Notably, we have however replicated the effects of HAL on cerebral morphometry observed in these same animals (Vernon *et al.* 2011) in a larger sample size of *n* = 12 per group (Vernon *et al.* 2012). We tested only one dose of each antipsychotic drug, which although within the range of striatal D_2_ receptor occupancy similar to levels obtained in clinical settings (Kapur *et al.* 2003) a full dose response study may be beneficial.

In conclusion, we show for the first time that chronic treatment of naive adult rats with either HAL or OLZ lead to changes in hippocampal shape metrics, in the absence of volume changes. Strikingly, the effects of both drugs were relatively common, particularly in the ventral hippocampus. Nevertheless, our data also suggest preliminary evidence for differential shape changes following HAL or OLZ exposure in the dorsal hippocampus, which require confirmation in a larger sample size. The effects were more profound with HAL, as compared to OLZ, consistent with recent clinical observations. It is however, important to stress that these data were collected in normal rats, which does not reflect the innate pathology of SCZ. Furthermore, because the cellular basis and functional consequences of these effects remain unknown, one should be cautious in drawing clinical inferences. Nonetheless, our studies provide a clear rationale for future experiments to explicitly examine the relationships between changes in hippocampal shape metrics and how these relate to the efficacy of APD as well as morphological, cognitive and metabolic side effects of chronic APD treatment.

## Supplementary material

For supplementary material accompanying this paper visit http://dx.doi.org/10.1017/S0033291716001768. click here to view supplementary material
