# Supplementary material for: Chronic exposure to haloperidol and olanzapine leads to common and divergent shape changes in the rat hippocampus in the absence of grey-matter volume loss
Source: Psychol Med. 2016 Aug 12;46(15):3081–93. doi: 10.1017/S0033291716001768 (PMC5108303; doi:10.1017/S0033291716001768)
Supplement: Supplementary file 1 [file S0033291716001768sup001.docx]

**Chronic treatment with haloperidol and olanzapine leads to common and divergent shape changes in the rat hippocampus in the absence of grey matter volume loss**

***Supplementary information***

**Supplementary Material and Methods**

**Antipsychotic Drug (APD) Administration**

Animals were randomly divided into three groups: 1) common vehicle (*n* = 8) (β-hydroxypropylcyclodextrin, 20% w/v, acidified by ascorbic acid, to pH 6); 2) 2 mg/kg/day haloperidol (HAL, *n*=8, Sigma-Aldrich, Dorset, UK); and 3) 10 mg/kg/day olanzapine (OLZ, *n*=8, Biophore Pharmaceuticals Ltd, Hyderabad, Andra Pradesh, India). Vehicle or drugs were administered subcutaneously from the 10th week of life, using MRI-safe osmotic minipumps (Alzet Model 2ML4, 28 days; Alzet, Cupertino, CA, USA) for 8 weeks. The doses of each APD were chosen based on previous D_2_ receptor occupancy studies ([Kapur *et al.*, 2003](#_ENREF_9), [Turrone *et al.*, 2003](#_ENREF_11)); serum plasma levels achieved following chronic administration in this study reflect D2 occupancy in the range of 75% to 90% ([Kapur *et al.*, 2003](#_ENREF_9), [Turrone *et al.*, 2003](#_ENREF_11)). This dosing regimen was tailored to capture clinical practice, i.e., OLZ with a median dose of 15 mg and HAL often in the range of 5 to 10 mg ([Vernon *et al.*, 2011](#_ENREF_12)). The results may have been different if the minimal therapeutic dose (i.e., 10 mg of OLZ or 2–3 mg of HAL) were modeled. The osmotic pump delivers at a steady rate in comparison with daily injections where drug levels fall to undetectable levels in 24 hours (half-life <2.5 hours in rats for most antipsychotics). Osmotic minipumps filled with drug or vehicle solutions were inserted subcutaneously on the back flank under isoflurane anesthesia (5% induction, 1.5% maintenance) and replaced once after 28 days. A blood sample was collected at termination for estimation of drug levels, done commercially using tandem mass spectrometry, revealing drug plasma levels of HAL and OLZ to be 20.58 ± 1.99 ng/mL and 60.13 ± 20.75 ng/mL (mean ± SD), respectively ([Vernon *et al.*, 2011](#_ENREF_12)).

**MRI: Brain preparation and acquisition**

Vehicle (*n*=8), HAL (*n*=8) and OLZ (*n*=8) treated animals were deeply anaesthetized with sodium pentobarbital (60 mg/kg, i.p.) and perfused transcardially with heparinized 0.9% saline followed by 4% paraformaldehyde (PFA). The heads were removed by decapitation and were allowed to postfix in 4% PFA at 4°C for 24 hr. The heads were then rinsed in 0.1 mol/L phosphate buffered saline containing 0.05% sodium azide, and stored in a fresh volume of this solution at 4°C for 5 weeks prior to imaging. Brains were imaged within skulls to minimize geometric distortion. Prior to imaging, samples were removed from storage solution, blotted, and placed into plastic snap-lock bags and inserted into a custom-built head radiofrequency coil with 43 mm internal diameter (Rapid Biomedical GmbH, Germany). Parameters used to acquire the scans were: a coronal *T*_2_-weighted, 2D multi-echo, multi-slice spin-echo (MEMS) pulse sequence consisting of 8 echoes, with TR/TE = 4200/10 msec, 4 averages, field-of-view 192 x 192 mm^3^, and matrix size = 35 x 35 mm, with 50 slices of 0.5 mm thickness, giving images with an in-plane resolution of 182 μm. MR images were converted off-line to NIFTI file format for TBM and shape analysis, respectively.

**Tensor Based Morphometry analysis**

All brains in the study were first rigidly aligned into a common coordinate system (the mean of all 24 scans) using an automated intensity-based group-wise registration approach ([Crum *et al.*, 2013a](#_ENREF_2), [Crum *et al.*, 2013b](#_ENREF_3)). A high-dimensional non-rigid registration algorithm was applied to warp each globally aligned scan to the reference ([Crum *et al.*, 2013a](#_ENREF_2), [Crum *et al.*, 2005](#_ENREF_4), [Fox *et al.*, 2001](#_ENREF_5)). Maps of localized volume difference at each voxel relative to the reference brain were computed from the log of the Jacobian determinant of this non-rigid transformation for each scan. Voxel-wise statistical tests over a hippocampal mask were performed to establish regions of significant volumetric difference between groups. This analysis was specifically constrained to the hippocampus since this was our *a priori* structure of interest ([Vernon *et al.*, 2011](#_ENREF_12)). Differences between groups were compared using a *t* statistic (2-tailed, unequal variance assumed). Permutation testing was used to establish statistical significance ([Bullmore *et al.*, 1999](#_ENREF_1)). Correction for multiple comparisons was performed using the false discovery rate thresholded at *q*=0.05 ([Genovese *et al.*, 2002](#_ENREF_6)).

**Post-mortem tissue preparation**

After the MRI acquisition, the brains were removed from the skull and immersion fixed in 4% PFA in 0.1 mol/L phosphate buffer, pH 7.4 for 1 day and then cryoprotected in 30% sucrose, 0.5% sodium azide in 0.1 mol/L phosphate buffer for 48 hours before storage in tissue cryoprotection solution (TCS; 25% glycerin (v/v) 30% ethylene glycol (v/v), 0.5% sodium azide in 0.2 M phosphate buffer) at -20°C. Serial coronal sections through the rostrocaudal extent of the brain were collected and stored in TCS at -20°C. To visualize neuronal cytoarchitecture, every 12^th^ section of each brain (8 rats from each treatment group) was mounted on gelatine/chrome alum-coated Superfrost microscope slides (VWR, Poole, UK), air dried overnight and stained for 30 min at 60°C with cresyl fast violet solution (0.05% solution with 0.5 ml of 10% acetic acid per 100 ml of solution were mixed and preheated to 56°C directly before use). Slides were next rinsed in distilled water and differentiated through a graded series of alcohol solutions (70, 90, 100%, 5 minutes in each). Sections were then cleared in 100% xylene and coverslipped with DPX (VWR) ([Pontikis *et al.*, 2004](#_ENREF_10)).

**Stereological assessment of hippocampus volume**

To verify our MRI analysis *post-mortem*, stereological analysis was carried out blinded to treatment group. An Olympus microscope with charge-coupled device camera and X-Y-Z motorized specimen stage (Olympus UK Ltd., Southend-on-Sea, Essex, United Kingdom) connected to a personal computer running *StereoInvestigator* software v7.0 (MBF BioScience, Williston, Vermont, USA) was used. A contour was drawn around the gray matter comprising the dorsal and ventral hippocampus combined in the left-brain hemisphere at ×2.0 magnification. Measurements of the hippocampus were made from seven consecutive sections (1 in 12 interval) covering approximately to -1.92 to -6.84 mm from bregma. A grid of points (200 x 200 µm) was superimposed over each section and all the points lying within the region-of-interest recorded automatically by the software. The average number of points hitting the hippocampus in each group were: vehicle, 241±18, haloperidol 223±19 and olanzapine 213±18. From these counts, using the Cavalieri estimator ([Gundersen and Jensen, 1987](#_ENREF_7)), the volume of each region in cubic millimeters was estimated using: *V=TaΣP_i_,* where *T* is the mean slice thickness; *a* is the area per point; and *P_i_* is the number of points falling upon the marked region. Coefficients of error were calculated with values <0.05 accepted ([Gundersen and Jensen, 1987](#_ENREF_7), [Gundersen *et al.*, 1999](#_ENREF_8)).

**Supplementary Results**


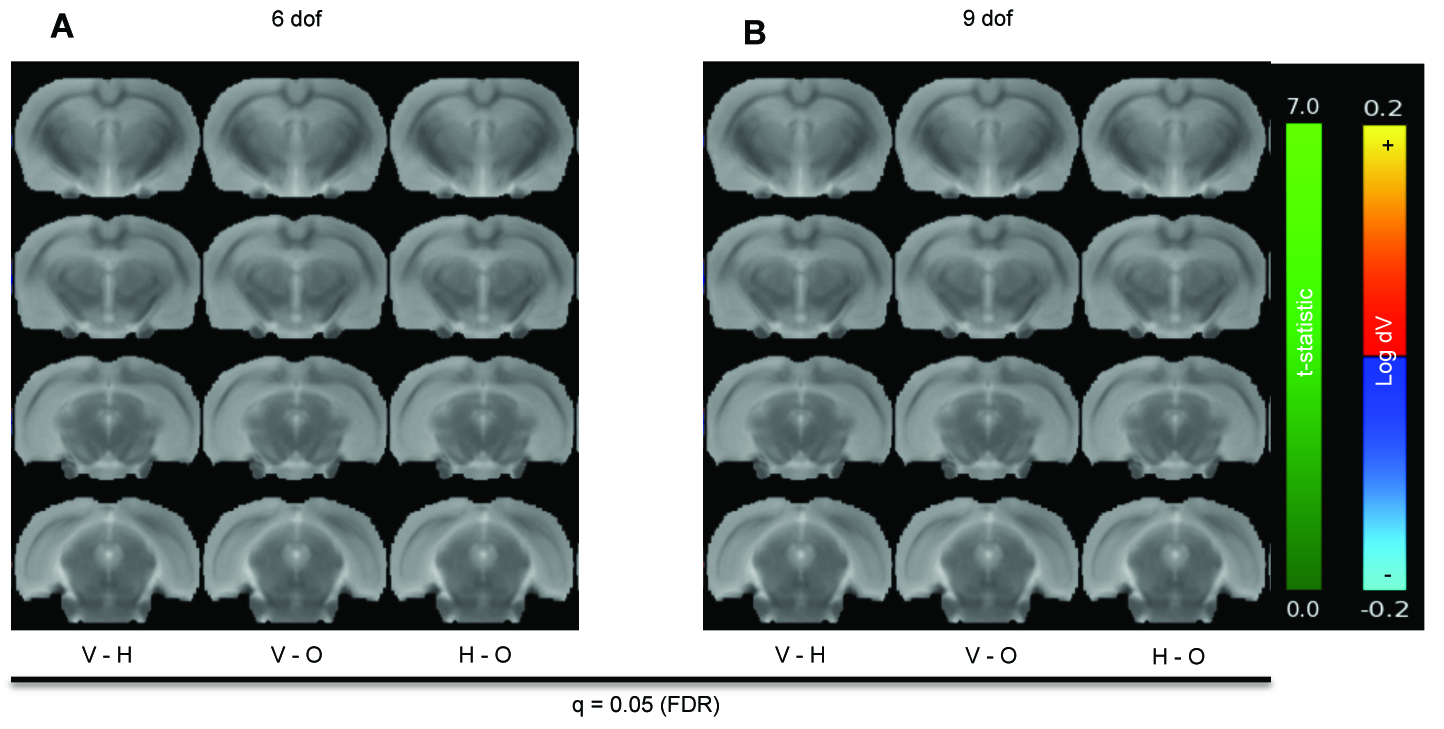


**Supplementary Figure S1.** TBM statistical maps demonstrating the absence of local volume differences in the hippocampus between vehicle (V), haloperidol (H) and olanzapine (O)-treated rats using either **(A)** 6 degrees of freedom or **(B)** 9 degrees of freedom in the non-linear registration to account for global scaling differences. Data were analyzed per voxel using the hippocampus binary mask shown in Figure 1. Data shown are FDR corrected (q=0.05). No significant clusters of volume change were found even at exploratory thresholds (p<0.05 uncorrected for multiple comparisons; *data not shown*).

| *Treatment group* | | *Left hippocampus volume (CE)* |
| --- | --- | --- |
| Vehicle | |  |
| Mean | 46.36 (0.009) | |
| SD | 4.02 | |
| CV | 0.08 | |
|  |  | |
| Haloperidol |  | |
| Mean | 41.77 (0.01) | |
| SD | 5.51 | |
| CV | 0.13 | |
|  |  | |
| Olanzapine |  | |
| Mean | 40.17 (0.01) | |
| SD | 8.01 | |
| CV | 0.20 | |

**Supplementary Table S1:** The total volume of the left hippocampus (dorsal + ventral) measured from Nissl stained *post-mortem* brain tissue sections obtained from rats exposed to vehicle, haloperidol (2 mg/kg/day s.c.) or olanzapine (10 mg/kg/day s.c.) for 8 weeks, using the Cavalieri probe. CV, coefficient of variation; CE, Gundersen Coefficient of error (m=1).

**References**

**Bullmore, E. T., Suckling, J., Overmeyer, S., Rabe-Hesketh, S., Taylor, E. & Brammer, M. J.** (1999). Global, voxel, and cluster tests, by theory and permutation, for a difference between two groups of structural MR images of the brain. *IEEE Transactions on Medical Imaging* **18**, 32-42.

**Crum, W. R., Giampietro, V. P., Smith, E. J., Gorenkova, N., Stroemer, R. P. & Modo, M.** (2013a). A Comparison of Automated Anatomical-Behavioural Mapping Methods in a Rodent Model of Stroke. *Journal of Neuroscience Methods* **219**(1), 27-40.

**Crum, W. R., Modo, M., Vernon, A. C., Barker, G. J. & Williams, S. C.** (2013b). Registration of challenging pre-clinical brain images. *Journal of Neuroscience Methods* **216**(1), 62-77.

**Crum, W. R., Tanner, C. & Hawkes, D. J.** (2005). Anisotropic multi-scale fluid registration: evaluation in magnetic resonance breast imaging. *Physics in Medicine and Biology* **50**, 5153-5174.

**Fox, N. C., Crum, W. R., Scahill, R. I., Stevens, J. M., Janssen, J. C. & Rossor, M. N.** (2001). Imaging of onset and progression of Alzheimer's disease with voxel-compression mapping of serial magnetic resonance images. *Lancet* **358**, 201-205.

**Genovese, C. R., Lazar, N. A. & Nichols, T.** (2002). Thresholding of statistical maps in functional neuroimaging using the false discovery rate. *Neuroimage* **15**, 870-878.

**Gundersen, H. J. & Jensen, E. B.** (1987). The efficiency of systematic sampling in stereology and its prediction. *Journal of Microscopy* **147**, 229-263.

**Gundersen, H. J., Jensen, E. B., Kieu, K. & Nielsen, J.** (1999). The efficiency of systematic sampling in stereology--reconsidered. *Journal of Microscopy* **193**, 199-211.

**Kapur, S., VanderSpek, S. C., Brownlee, B. A. & Nobrega, J. N.** (2003). Antipsychotic dosing in preclinical models is often unrepresentative of the clinical condition: a suggested solution based on in vivo occupancy. *Journal of Pharmacology and Experimental Therapeutics* **305**, 625-631.

**Pontikis, C. C., Cella, C. V., Parihar, N., Lim, M. J., Chakrabarti, S., Mitchison, H. M., Mobley, W. C., Rezaie, P., Pearce, D. A. & Cooper, J. D.** (2004). Late onset neurodegeneration in the Cln3-/- mouse model of juvenile neuronal ceroid lipofuscinosis is preceded by low level glial activation. *Brain Research* **1023**, 231-242.

**Turrone, P., Remington, G., Kapur, S. & Nobrega, J. N.** (2003). The relationship between dopamine D2 receptor occupancy and the vacuous chewing movement syndrome in rats. *Psychopharmacology (Berlin)* **165**, 166-71.

**Vernon, A. C., Natesan, S., Modo, M. & Kapur, S.** (2011). Effect of chronic antipsychotic treatment on brain structure: a serial magnetic resonance imaging study with ex vivo and postmortem confirmation. *Biological Psychiatry* **69**, 936-944.
